# Case report: Revealing the rare—a Brody Disease patient from Turkey expanding the phenotype

**DOI:** 10.3389/fgene.2023.1289312

**Published:** 2023-11-30

**Authors:** Ayça Şahin, Esmer Zeynep Duru Badakal, Müge Kovancılar Koç, Hilmi Uysal, Ayşe Nazlı Başak

**Affiliations:** ^1^ Suna and İnan Kıraç Foundation, Neurodegeneration Research Laboratory, Koç University Research Center for Translational Medicine (KUTTAM), School of Medicine, Koç University, İstanbul, Türkiye; ^2^ Department of Neurology, Faculty of Medicine, Akdeniz University, Antalya, Türkiye

**Keywords:** Brody Disease, Brody Myopathy, *ATP2A1*, SERCA1, rare disease, whole-exome sequencing

## Abstract

Brody Disease is an exceptionally rare, autosomal recessive myopathy attributed to the pathogenic variants in the *ATP2A1*, which encodes the sarcoplasmic/endoplasmic reticulum Ca (2+) ATPase type 1 protein SERCA1. It was first described by Brody IA in 1969. To date, only thirty-three Brody families with forty-seven patients have been reported in the literature, and the disease prevalence is considered as 1 in 10 million, demonstrating the peculiarity of the disease. Clinical characteristics of Brody Disease include muscle stiffness after exercise, myalgia, and muscle cramps. Brody Disease patients generally have disease onset in the first decade, and genetic diagnosis is delayed as a consequence of both the rareness and the mild course of the disease. Here, we report a Turkish Brody Disease patient with a homozygous c.428G>A p.Arg143Gln (NM_004320.4) missense mutation in the *ATP2A1*. The male patient, whose symptoms started at the age of 14–15, is now 36 years old. His clinical manifestations are athletic appearance, exotropia, slightly elevated creatine kinase (CK), mild progressive proximal muscle weakness in the lower extremities, muscle cramps, pain and stiffness. The patient described here has a very mild progression with an onset in the second decade, expanding the Brody Disease phenotype. The study also implies that in the era of emerging genetic therapies, the routine testing of patients with myopathies is a prerequisite since not only future therapies will be designed on molecular findings, but also currently available symptomatic and palliative treatment options will be more precisely applied.

## 1 Introduction

While rare diseases are classified as diseases affecting less than 1 in 2000 people, three major classifications exist: more common, less common, and ultra-rare ones. With a prevalence, estimated as 1 in 10 million, Brody Disease is one of the ultra-rare diseases. To our knowledge, only 33 Brody families with 47 patients have been reported in the literature so far (Braz et al., 2019; Bruels et al., 2019; Molenaar et al., 2020; Brugnoni et al., 2021; Saat and Sahin, 2021; Velardo et al., 2023; Walia and Su, 2023).

Brody Disease is an autosomal recessive myopathy caused by pathogenic variants in the *ATP2A1* (ATPase sarcoplasmic/endoplasmic reticulum Ca^2+^ transporting 1) (OMIM: # 108730) in which patients experience muscle stiffness after exercise, myalgia, and muscle cramps (Molenaar et al., 2020).

The underlying pathology of the disease arises from the difficulty in removing Ca^2+^ from intracellular space for muscle relaxation. The motor unit, composed of motor neurons and muscle fibers, is the core region of muscle movement, and skeletal muscles are innervated by alpha motor neurons. For a healthy movement, there is a well-established balance between the contraction and relaxation of the muscles. At the physiological level, the synaptic connection between the motor neuron and muscle is called the neuromuscular junction, in which the neurotransmitter acetylcholine is released from the alpha motor neuron, leading to the formation of an action potential that initiates muscle contraction. At the cellular level, upon the transmission of the action potential, Ca^2+^ is released from the sarcoplasmic reticulum, increasing the [Ca^2+^] level in the intracellular space, leading to muscle contraction. Relaxation, on the other hand, occurs once the Ca^2+^ is pumped back to the sarcoplasmic reticulum. Transportation of Ca^2+^ to the sarcoplasmic reticulum is performed by a calcium pump named SERCA (sarcoplasmic endoplasmic reticulum calcium ATPase). SERCA, which is the most abundant protein in the sarcoplasmic reticulum, hydrolyzes ATP per two molecules of Ca^2+^ transported (Koeppen and Stanton, 2023).

SERCA1, a muscle-specific protein, has two splice variants, SERCA1a, present in adults, and neonatal SERCA1b, containing 994 and 1,001 aminoacids, respectively (Wuytack et al., 2002; Xu and Van Remmen, 2021). It is mainly expressed in fast-twitch muscles (Xu and Van Remmen, 2021), also called type 2 muscle fibers (Molenaar et al., 2020). *ATP2A1* encodes SERCA1 protein, which plays a role in muscle relaxation by removing Ca^2+^ from the cytosol (Guglielmi et al., 2013).

The first patient described by Brody in 1969 was a 26-year-old male patient with the prominent symptom of muscle stiffness, especially after strenuous and rapid activities. The patient had no family history of the disease; his first memory was an unexplained fall at the age of five as his muscles got stiffened during a foot race (Brody, 1969).

Here, we report a Turkish male patient with Brody Disease whose parents are first-degree cousins.

## 2 Case presentation

The patient is a 36-year-old male, referred to the Neurodegeneration Research Laboratory, KUTTAM, Koç University Hospital for genetic analysis from the Neurology Department of Akdeniz University Hospital School of Medicine. He is the second child of first-degree consanguineous parents. His initial complaints started at the age of 14–15, with severe muscle pain while playing football. The current symptoms of the patient with an athletic appearance include mild progressive muscle weakness in lower extremities, left prominent bilateral exotropia, cramps, pain and stiffness in muscles, cold-induced myalgias, and an elevated creatine kinase (CK: 630 IU/L). The MRC sum score is 54 (N = 60). Moderate myopathic changes were observed in the lower extremity proximal muscles on EMG. Silent cramps were not observed, and there was not a significant change in repetitive nerve stimulation. Spontaneous denervation potentials or myotonic discharges were not observed. Sensory and motor conduction velocities were within normal limits. The alpha-glucosidase level was found to be normal. The patient was started on steroids 2 years before the genetic diagnosis. Although the treatment provided some relief from his symptoms, a hip prosthesis was implanted due to aseptic necrosis of the femoral head.

## 3 Methods

Informed written consent was obtained from the patient as well as from his family members. Genomic DNA was isolated from peripheral blood using Qiagen EZ1 Advanced XL. Whole exome sequencing was performed (Macrogen, Amsterdam) for the index case. WES raw data was processed using the SEQ Platform (Genomize, Istanbul, Turkey), and the data was analyzed at NDAL. The following filtering criteria were applied: OMIM-related genes, minor allele frequency (MAF) < 1%, destructive, missense, and splice region variants. The pathogenicity of the homozygous *ATP2A1* variant detected was evaluated by *in silico* tools. Subsequently, segregation analysis using Sanger sequencing was performed. The pathogenic variant was numbered according to the transcript NM_004320.4, and was submitted to ClinVar (https://www.ncbi.nlm.nih.gov/clinvar/variation/464089/Accession: SCV004023391.1).

## 4 Results

### 4.1 Genetic analysis

**FIGURE 1:**
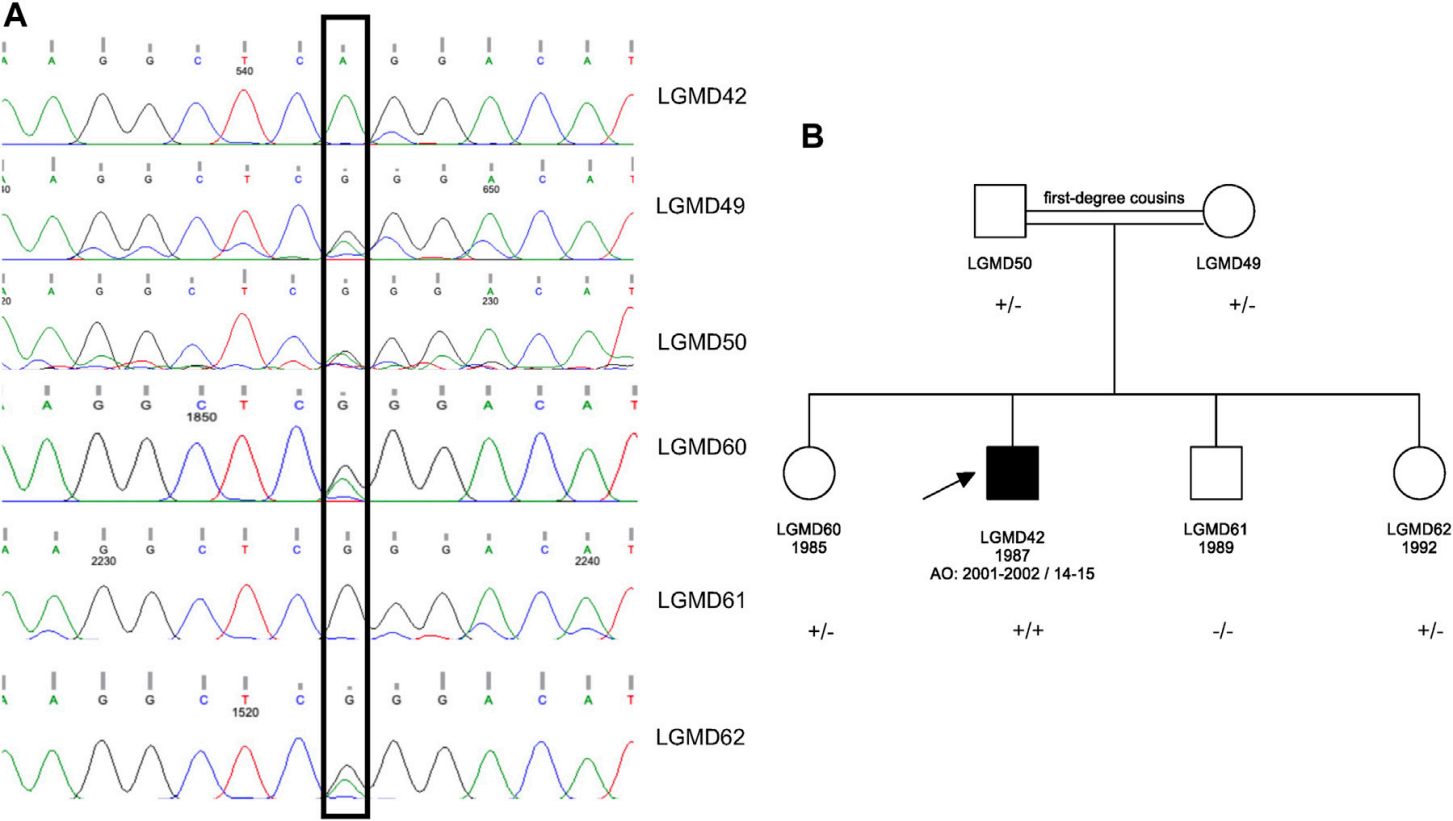
**(A)** Sanger sequencing results for the proband and his asymptomatic family members. **(B)** Pedigree of the family. The pathogenic variant is *ATP2A1* (NM_004320.4) p.Arg143Gln in Exon 5; +: mutant allele and -: wild type allele.

WES analysis revealed a homozygous c.428G>A p.Arg143Gln variant in the *ATP2A1*. This variant was earlier reported in a patient with Brody Disease in a compound heterozygous state with c.1317_1318del p.Glu439Aspfs*80 in trans (Molenaar et al., 2020) (Table 1). Sanger sequencing was performed for the proband and his asymptomatic family members. The variant was validated in the proband in homozygous dosage and in his parents in heterozygous form. Two siblings were also found to be carriers, and one sibling was wild type (Figure 1).

**TABLE 1:** The comparison between Molenaar’s patient and the Turkish patient under study.

Clinical and genetic features	Molenaar et al. (2020) patient	Turkish patient
Genotype	Compound Heterozygous	Homozygous
Pathogenic Variant	c.428G>A in trans with c.1317_1318del	c.428G>A
Symptom Onset	Third Decade	Second Decade
Age at Diagnosis	51 years old	36 years old
Creatine Kinase (CK)	300–600 IU/L	630 IU/L
Athletic appearance	Yes	Yes
Involvement pattern	upper limbs and lower limbs	lower extremity predominance
Delayed relaxation of eyelids	No	No, but the patient has left prominent bilateral exotropia
Muscle stiffness	Yes—at the start of exercise and during exercise	Yes
Muscle weakness	No	Yes—Proximal lower extremity (MRC score: 4/5)
Muscle hypertrophy	No	Unnoticeable
Muscle cramps	Yes—during exercise	Yes
Myalgia	Yes—during exercise and post-exercise	Yes
Delayed relaxation of muscles	Yes—delayed relaxation of upper limb after sustained contraction	Unnoticeable delayed relaxation after sustained contraction
Increase of symptoms in cold	Yes	Yes

### 4.2 *In silico* findings

The homozygous c.428G>A p.Arg143Gln variant identified in the actuator domain of the SERCA1 protein is a missense mutation. While several tools supported the pathogenicity, DANN: 0.999, CADD: 22.7000, FATHMM-MKL: 0.9749, a few remained uncertain or benign-supporting. Conservation scores suggested a conserved site with PhyloP100way: 7.585, PhastCons100way: 1.000, and GERP RS: 4.1799. In our cohort consisting of 1740 WES-subjected individuals from Turkey, the variant is present in five independent cases in heterozygous state.

Notably, this missense mutation was already reported in trans position with Brody Disease in the compound heterozygous state with a frameshift mutation (Molenaar et al., 2020). In ClinVar (https://www.ncbi.nlm.nih.gov/clinvar/), there were three entries for the variant, classifying it as VUS (variant of uncertain significance). In GnomAD (https://gnomad.broadinstitute.org/), the variant was found in 33 individuals in the heterozygous state, no individual had the variant in homozygous state, and the reported allele frequency was 0.0001168. The variant was reported as likely pathogenic in the LOVD database (https://www.lovd.nl/).

## 5 Discussion

Since Brody’s identification of the disease 54 years ago, only 47 patients in 33 families have been identified, demonstrating the extreme rareness of the disease. Here, we define the clinical and genetic findings in a Turkish Brody Disease patient.

In general, the first symptoms of Brody Disease manifest in the first decade; however, patients may not feel the urge to consult a physician for several years. In the literature, patients described so far usually have the onset during childhood. Still, their diagnosis is delayed for several years, presumably due to the mild progression of the disease, as well as its rarity and thus ignorance. Also, in our patient, from the first symptoms (describing severe muscle pain at the age of 14–15 while playing football) to the molecular diagnosis, more than 20 years have passed. Owing to the mild course of the disease, the patient describes his complaints as being around for 4–5 years only. However, his first diagnosis was initially put around the age of 18 when he was excluded from the military service because of his muscle-related complaints. Yet, another 16 years had to pass until the patient was referred to our center for molecular testing with the preliminary diagnosis of Limb Girdle Muscular Dystrophy (LGMD). His muscle disease was reported as mildly progressive, with major complaints in his knees, ankles, and hips. The patient has undergone femur surgery, he now has a one-sided hip prosthesis. Although malignant hyperthermia was observed in a few Brody Disease patients (Molenaar et al., 2020), our patient did not manifest malignant hyperthermia during the surgery.

The missense mutation identified (c.428G>A; p.Arg143Gln) is in the actuator domain of the protein. Eight mutations in 10 families comprising 15 patients have been reported in this domain so far (10 index cases and 5 affected siblings). Twelve out of fifteen had an onset in the childhood/first decade, and there was a male predominance. Demographic and clinical characteristics of all patients with a mutation in the actuator domain are compiled in Table 2.

**TABLE 2:** Genetic and clinical information of patients with mutations in the actuator domain.

Family number	Patient number	Gender	Ethnicity	Age at onset (decade)	Age at diagnosis	Genotype	DNA sequence change	Aminoacid change	Mutation type	Clinical information	References
1	1	F	Italian	first	not known	Compound heterozygous	c.100G>T *in trans with c.1167C>A*	p.Glu34* Exon 1 in trans with p.Tyr389* *Exon 10*	Nonsense *Nonsense*	- Involvement Pattern: UL + LL + F + E	Molenaar et al. (2020) Odermatt et al. (2000)
										- Muscle stiffness during exercise	
										- Mild proximal muscle weakness	
										- Delayed relaxation of upper limb after sustained contraction	
										- Increase of symptoms in cold	
										- Athletic appearance	
										- EMG performed: Silent contractures	
2	2	M	-	first	8	Homozygous	c.100G>T	p.Glu34* Exon 1	Nonsense	- Involvement Pattern: UL + LL + E	Molenaar et al. (2020)
										- Muscle stiffness during exercise	
										- Myalgia during exercise and post-exercise	
										- Delayed relaxation of upper limb after sustained contraction	
										- Delayed relaxation of eyelids after repetitive as well as after sustained contraction	
										- Increase of symptoms in cold	
										- Athletic appearance	
										- EMG performed	
										- CK (IU/L):93	
	3	M	-	first	6					- Involvement Pattern: UL + LL	
										- Muscle stiffness during exercise	
										- Myalgia during exercise and post-exercise	
										- Delayed relaxation of upper limb after repetitive as well as after sustained contraction	
										- Delayed relaxation of eyelids after repetitive as well as after sustained contraction	
										- Increase of symptoms in cold	
										- Athletic appearance	
										- EMG performed	
3	4	M	-	third	51	Compound heterozygous	c.428G>A *in trans with c.1317_1318del*	p.Arg143Gln Exon 5 in trans with p.Glu439Aspfs*80 *Exon 12*	Missense *Frameshift*	- Involvement Pattern: UL + LL	Molenaar et al. (2020)
										- Muscle stiffness at the start of exercise and during exercise	
										- Muscle cramps during exercise	
										- Myalgia during exercise and post-exercise	
										- Delayed relaxation of upper limb after sustained contraction	
										- Increase of symptoms in cold	
										- Athletic appearance	
										- EMG performed	
										- CK (IU/L): 300-600	
**4**	**5 (LGMD42)**	**M**	**Kurdish**	**second**	**36**	**Homozygous**	**c.428G>A**	**p.Arg143Gln** **Exon 5**	**Missense**	**- Involvement Pattern: Lower extremity predominance**	**This study**
										**- Muscle stiffness**	
										**- Muscle pain**	
										**- Muscle weakness (Proximal lower extremity: 4/5)**	
										**- Unnoticeable muscle hypertrophy**	
										**- Unnoticeable delayed relaxation after sustained contraction**	
										**- Left prominent bilateral exotropia**	
										**- Increase of symptoms in cold**	
										**- Athletic appearance**	
										**- EMG performed**	
										**- CK (IU/L): 630**	
5	6	M	-	first	42	Homozygous	c.440del	p.Pro147Leufs*34 Exon 5	Frameshift	- Involvement Pattern: UL + LL	Molenaar et al. (2020) Karpati et al. (1986)
										- Muscle stiffness during exercise	
										- Muscle cramps	
										- Myalgia during exercise	
										- Mild proximal muscle weakness	
										- Delayed relaxation of upper limb after sustained contraction	
										- EMG performed	
										- CK (IU/L): 50	
	7	M	-	first	not known					- Involvement Pattern: UL + LL	
										- Muscle stiffness during exercise	
										- Muscle cramps	
										- Delayed relaxation of upper limb after repetitive contraction	
										- EMG performed	
										- CK (IU/L): 62	
6	8	M	-	first	32	Homozygous	c.490C>T	p.Arg164* Exon 6	Nonsense	- Involvement Pattern: UL + LL + F + E	Molenaar et al. (2020)
										- Muscle stiffness during exercise	
										- Delayed relaxation of upper limb after repetitive as well as after sustained contraction	
										- Delayed relaxation of eyelids after repetitive as well as after sustained contraction	
										- Increase of symptoms in cold	
										- Athletic appearance	
										- CK (IU/L): 219	
7	9	F	-	first	not known	Homozygous	c.547G>A	p.Glu183Lys Exon 7	Missense	- Involvement Pattern: UL + LL + F + E	Molenaar et al. (2020)
										- Muscle stiffness during exercise	
										- Increase of symptoms in cold	
										- EMG performed: Silent contractures	
8	10	M	Kurdish	first	41	Homozygous	c.592C>T	p.Arg198* Exon 7	Nonsense	- Involvement Pattern: UL + LL + F + E	Molenaar et al. (2020) Benders et al. (1994) Odermatt et al. (1996)
										- Muscle stiffness and cramps during exercise	
										- Myalgia during exercise and post-exercise	
										- Muscle hypertrophy	
										- Delayed relaxation of upper limb after repetitive as well as after sustained contraction	
										- Delayed relaxation of eyelids after repetitive as well as after sustained contraction	
										- Increase of symptoms in cold	
										- Athletic appearance	
	11	M		first	42					- Involvement Pattern: UL + LL + F + E	
										- Muscle stiffness and cramps during exercise	
										- Muscle hypertrophy	
										- Delayed relaxation of upper limb after repetitive as well as after sustained contraction	
										- Delayed relaxation of eyelids after repetitive as well as after sustained contraction	
										- Increase of symptoms in cold	
										- Athletic appearance	
	12	M		first	47					- Involvement Pattern: UL + LL + F + E	
										- Muscle stiffness and cramps during exercise	
										- Myalgia during exercise	
										- Rare, mild, or minor myalgia post-exercise	
										- Muscle hypertrophy	
										- Delayed relaxation of upper limb after repetitive as well as after sustained contraction	
										- Delayed relaxation of eyelids after repetitive contraction	
										- Increase of symptoms in cold	
										- Athletic appearance	
9	13	M	Italian	not known	28	Homozygous	c.623T>C	p.Leu208Pro Exon 7	Missense	- Muscle stiffness	Brugnoni et al. (2021)
10	14	M	-	first	not known	Compound heterozygous	c.704T>A	p.Ile235Asn Exon 8	Missense	- Involvement Pattern: LL	Molenaar et al. (2020) Sambuughin et al. (2014)
										- Myalgia in rest and post-exercise	
										- Muscle weakness	
										- Muscle pain	
										- Delayed relaxation of eyelids after repetitive contraction	
										- Increase of symptoms in cold	
										- Athletic appearance	
										- CK (IU/L): 183	
	15	F	-	first	not known		*in trans with c.2944G>A*	*in trans with p.Glu982Lys* *Exon 21*	*Missense*	- Myalgia	
										- Muscle weakness	
										- Muscle pain	
										- CK: Normal	

Table 1: Mutations are based on transcript: NM_004320.4.

Patient 5 is the case presented in this study, written in bold.

The mutations in trans not located in the actuator domain are written in italics.

UL, upper limbs; LL, lower limbs; F, facial; E, eyelids; CK, creatine kinase.

**FIGURE 2:**
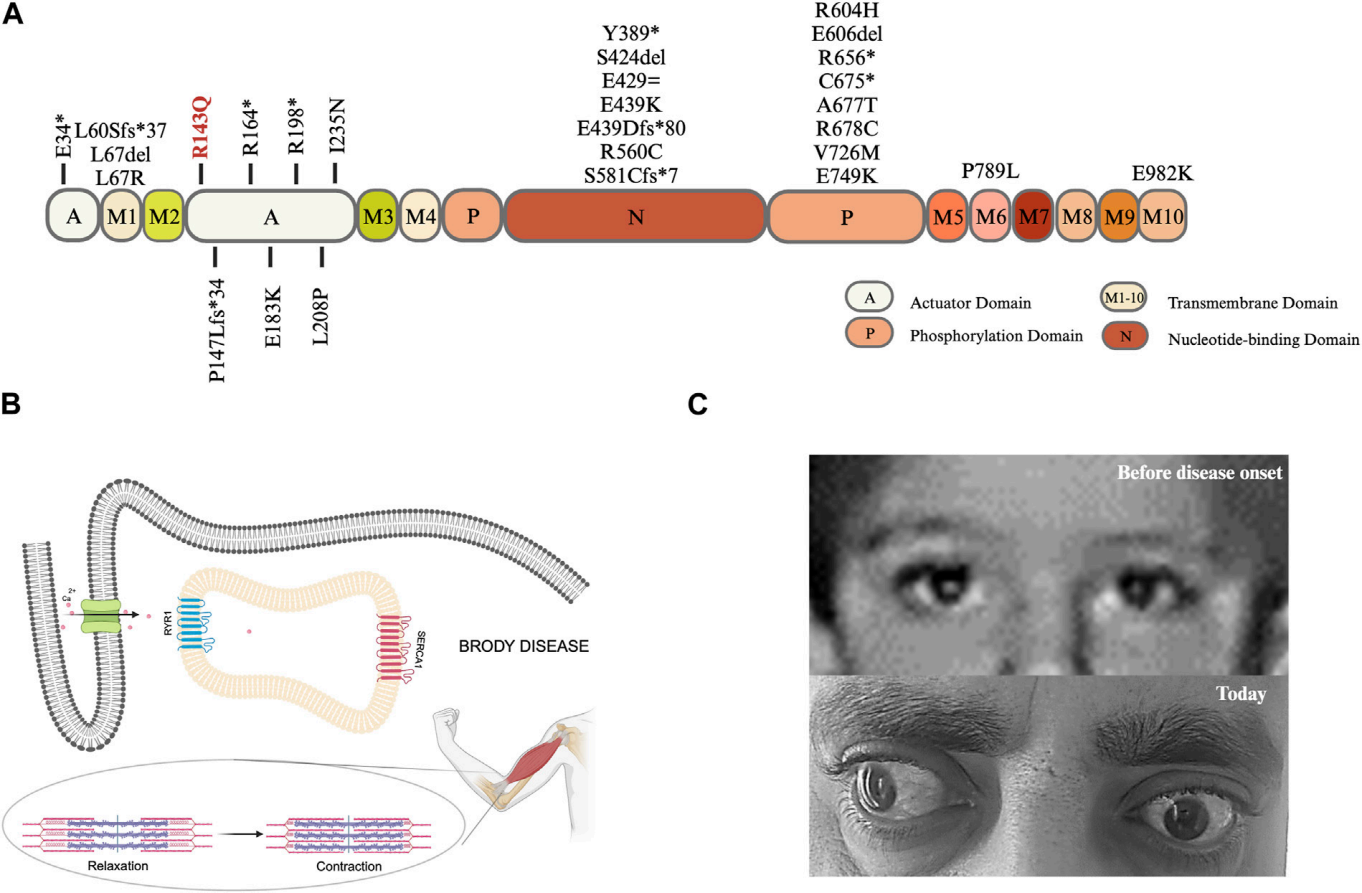
**(A)** Lolliplot diagram: SERCA1a is a transmembrane protein with a molecular weight of 110 kDa. The protein consists of a transmembrane (M) domain and a headpiece within the cytoplasm containing three domains: i) actuator **(A)** domain accounting for the dephosphorylation ii) phosphorylation (P) domain harboring a site for autophosphorylation, and iii) nucleotide-binding (N) domain which is the ATP binding site (Wuytack et al., 2002; Barbot et al., 2021), created with BioRender.com. **(B)** Graphical illustration of cellular and physiological mechanisms in Brody Disease. Adapted from Molenaar et al., 2016; Braz et al., 2019, created with BioRender.com. **(C)** The pictures of patient’s eyes before and after disease onset.

In total, there are 41 distinct mutations in the *ATP2A1* in 34 families worldwide, including our family; 28 mutations located in the different domains of SERCA1 are shown in Figure 2A. The mutations seem to be rare and almost family-private. In the actuator domain, eight variants were identified in 10 index cases; only two of these were redundant. Out of 10 index cases with a mutation in the actuator domain, seven are homozygous, whereas, in three patients, it is in a compound heterozygous state.

So far, three Brody families from Turkey, including our case, with three different mutations have been described. All three families have distinct mutations. The first family is Kurdish, with six children, three of whom are affected. The two older brothers were described by Benders et al. (1994), and their pathogenic variant was identified by Odermatt et al. (1996) (c.592C>T; p.Arg198*). A 5-year-old male patient with muscle weakness and delayed development was reported by Saat and Sahin (2021) in a cohort study with hereditary myopathies (c.2029G>A; p.Ala677Thr). To the best of our knowledge, the present study is the first description of the p.Arg143Gln missense mutation in biallelic state. The patient originating from the Southeastern part of Turkey has Kurdish ethnicity. As in the above two families, the pathogenic variant is present in homozygous form, the result of close consanguinity, very commonly practiced in this part of the country.

The male patient defined by Molenaar et al. (2020) harbored the same variant (c.428G>A; p.Arg143Gln) in a compound heterozygous state in trans with c.1317_1318del; p.Glu439Aspfs*80. Similar to our patient and to other Brody Disease cases, there is a long diagnostic odyssey between symptom onset and definite diagnosis. Table 1 compares the demographic, clinical, and genetic features of both patients. In our patient, the earlier disease onset and higher CK level, compared to Molenaar’s patient, may be the result of the homozygous effect, e.g., double dosage of the variant, or other modifying factors may also have contributed.

Molenaar et al. (2020) reported exercise-induced muscle stiffness in all patients and increase of symptoms when exposed to cold in majority of patients. Our patient’s complaints are consistent with the reported findings, as he frequently expresses exercise-induced muscle stiffness and cold-induced myalgia. Muscle relaxation issues are commonly observed in Brody patients, which are related to the Ca^2+^ removal difficulty from intracellular space. While RYR1 (ryanodine receptor 1) works by releasing Ca^2+^, SERCA1 works by removing Ca^2+^, both are fundamental in muscle contraction and relaxation (Figure 2B).

Eyelid muscle can also be affected in Brody Disease patients as Molenaar et al. (2020) reported delayed relaxation of eyelids i) after repetitive contraction in 61% and ii) after sustained contraction in 59% of total known cases. Although our case does not experience delayed relaxation of eyelids, he has left prominent bilateral exotropia, which developed during the disease course (Figure 2C). Toğrul et al. (2021) conducted a study with the extraocular muscles of patients who had strabismus surgery vs. healthy controls (corneal transplant donors) aiming to compare calcium adenosine 5′-triphosphatase (Ca^2+^-ATPase) enzyme activity and found that the enzyme activity is lower in the strabismus surgery patients. This finding, as well as the healthy appearance of eyes before disease onset, could indicate that the detected exotropia in our patient might be related to Brody Disease.

Today, there are still no treatment options for most rare diseases, and this also includes Brody Disease. The two drugs, verapamil and dantrolene, with limited therapeutic benefit, work by inhibiting the RYR1 channel and the dihydropyridine receptor, but are not without side effects. Animal models are crucial to enlighten disease-related mechanisms and to discover potential therapeutic candidates. Akyurek et al. (2021) demonstrated Chianina cattle congenital pseudomyotonia as a true counterpart animal model for Brody Disease and a novel pharmacological approach was introduced by the same group.

In the era of gene-based/molecular therapies, a firm differential diagnosis using next-generation technologies as the gold standard is inevitable since not only currently available symptomatic and palliative treatment options will be more precisely applied but also current and future therapies will be designed on molecular findings. In the decade of emerging genetic therapies, the routine testing of patients with myopathies becomes a prerequisite and is not curiosity-driven research anymore. We have all reasons to be optimistic for our rare (disease) patients.

## Data Availability

The datasets for this article are not publicly available due to concerns regarding participant/patient anonymity. Requests to access the datasets should be directed to the corresponding author.

## Abbreviations

ATP2A1, ATPase sarcoplasmic/endoplasmic reticulum Ca^2+^ transporting 1; SERCA1, Sarcoplasmic/endoplasmic reticulum Ca (2+) ATPase type 1; CK, Creatine kinase; EMG, Electromyography; WES, whole-exome sequencing; LGMD, Limb Girdle Muscular Dystrophy; RYR1, ryanodine receptor 1; CFTR, Cystic Fibrosis Transmembrane Regulator.

## References

[B1] Akyurek E. E. Joana G. Marilena B. Angelo P. Sandona D. Arcangelo G. (2021). Frontiers in metabolic research, 37. https://hdl.handle.net/11577/3478066.Human Brody disease and its animal model cattle pseudomyotonia: from understanding the pathogenetic mechanism to identification of novel therapeutic approaches

[B2] Barbot T. Beswick V. Montigny C. Quiniou É. Jamin N. Mouawad L. (2021). Deciphering the mechanism of inhibition of SERCA1a by sarcolipin using molecular simulations. Front. Mol. Biosci. 7, 606254. 10.3389/fmolb.2020.606254 33614704 PMC7890198

[B3] Benders A. A. Veerkamp J. H. Oosterhof A. Jongen P. J. Bindels R. J. Smit L. M. (1994). Ca2+ homeostasis in Brody’s disease. A study in skeletal muscle and cultured muscle cells and the effects of dantrolene an verapamil. J. Clin. Investig. 94, 741–748. 10.1172/JCI117393 8040329 PMC296154

[B4] Braz L. Soares-dos-Reis R. Seabra M. Silveira F. Guimarães J. (2019). Brody disease: when myotonia is not myotonia. Pract. Neurol. 19, 417–419. 10.1136/practneurol-2019-002224 30996034

[B5] Brody I. A. (1969). Muscle contracture induced by exercise. A syndrome attributable to decreased relaxing factor. N. Engl. J. Med. 281, 187–192. 10.1056/NEJM196907242810403 4239835

[B6] Bruels C. C. Li C. Mendoza T. Khan J. Reddy H. M. Estrella E. A. (2019). Identification of a pathogenic mutation in ATP2A1 via *in silico* analysis of exome data for cryptic aberrant splice sites. Mol. Genet. Genomic Med. 7, e552. 10.1002/mgg3.552 30688039 PMC6418371

[B7] Brugnoni R. Maggi L. Canioni E. Verde F. Gallone A. Ariatti A. (2021). Next-generation sequencing application to investigate skeletal muscle channelopathies in a large cohort of Italian patients. Neuromuscul. Disord. 31, 336–347. 10.1016/j.nmd.2020.12.003 33573884

[B8] Guglielmi V. Voermans N. C. Gualandi F. Van Engelen B. G. Ferlini A. Tomelleri G. (2013). Fourty-four years of Brody disease: it is time to Review. J. Genet. Syndr. Gene Ther. 04. 10.4172/2157-7412.1000181

[B9] Karpati G. Charuk J. Carpenter S. Jablecki C. Holland P. (1986). Myopathy caused by a deficiency of Ca2+-adenosine triphosphatase in sarcoplasmic reticulum (Brody’s disease). Ann. Neurol. 20, 38–49. 10.1002/ana.410200108 2943216

[B10] Koeppen B. M. Stanton B. A. (2023). Berne and levy physiology e-book. Elsevier Health Sciences.

[B11] Molenaar J. P. Snoeck M. M. Voermans N. C. van Engelen B. G. (2016). Overactive muscles: it can be more serious than common myalgia or cramp. Ned. Tijdschr. Geneeskd. 160, A9675. https://hdl.handle.net/2066/165866 . 27122070

[B12] Molenaar J. P. Verhoeven J. I. Rodenburg R. J. Kamsteeg E. J. Erasmus C. E. Vicart S. (2020). Clinical, morphological and genetic characterization of Brody disease: an international study of 40 patients. Brain 143, 452–466. 10.1093/brain/awz410 32040565 PMC7009512

[B13] Odermatt A. Barton K. Khanna V. K. Mathieu J. Escolar D. Kuntzer T. (2000). The mutation of Pro789 to Leu reduces the activity of the fast-twitch skeletal muscle sarco(endo)plasmic reticulum Ca2+ ATPase (SERCA1) and is associated with Brody disease. Hum. Genet. 106, 482–491. 10.1007/s004390000297 10914677

[B14] Odermatt A. Taschner P. E. M. Khanna V. K. Busch H. F. Karpati G. Jablecki C. K. (1996). Mutations in the gene–encoding SERCA1, the fast–twitch skeletal muscle sarcoplasmic reticulum Ca2+ ATPase, are associated with Brody disease. Nat. Genet. 14, 191–194. 10.1038/ng1096-191 8841193

[B15] Saat H. Sahin I. (2021). Mutation spectrum of hereditary myopathies in Turkish patients and novel variants. Ann. Hum. Genet. 85, 178–185. 10.1111/ahg.12429 33963534

[B16] Sambuughin N. Zvaritch E. Kraeva N. Sizova O. Sivak E. Dickson K. (2014). Exome analysis identifies Brody myopathy in a family diagnosed with malignant hyperthermia susceptibility. Mol. Genet. Genomic Med. 2, 472–483. 10.1002/mgg3.91 25614869 PMC4303217

[B17] Toğrul V. Günaydın N. T. Tanyıldız B. (2021). CALCIUM ADENOSINE 5’ TRIPHOSPHATASE ENZYME ACTIVITY IN EXTRAOCULAR MUSCLES IN STRABISMUS. KTD 22, 93–97. 10.18229/kocatepetip.656268

[B18] Velardo D. Antognozzi S. Rimoldi M. Pagliarani S. Cogiamanian F. Barbieri S. (2023). Case report: clinical and molecular characterization of two siblings affected by Brody myopathy. Front. Neurol. 14, 1170071. 10.3389/fneur.2023.1170071 37332993 PMC10272758

[B19] Walia S. Su X. (2023). Brody myopathy associated with novel compound heterozygous ATP2A1 mutations (P6-8.015). Neurology 100. 10.1212/WNL.0000000000202716

[B20] Wuytack F. Raeymaekers L. Missiaen L. (2002). Molecular physiology of the SERCA and SPCA pumps. Cell. Calcium 32, 279–305. 10.1016/S0143416002001847 12543090

[B21] Xu H. Van Remmen H. (2021). The SarcoEndoplasmic Reticulum Calcium ATPase (SERCA) pump: a potential target for intervention in aging and skeletal muscle pathologies. Skelet. Muscle 11, 25. 10.1186/s13395-021-00280-7 34772465 PMC8588740

